# Supplementary figures and images for: Base editing of Ptbp1 in neurons alleviates symptoms in a mouse model of Parkinson’s disease
Source: eLife. 2024 Dec 23;13:RP97180. doi: 10.7554/eLife.97180 (PMC11666242; doi:10.7554/eLife.97180)

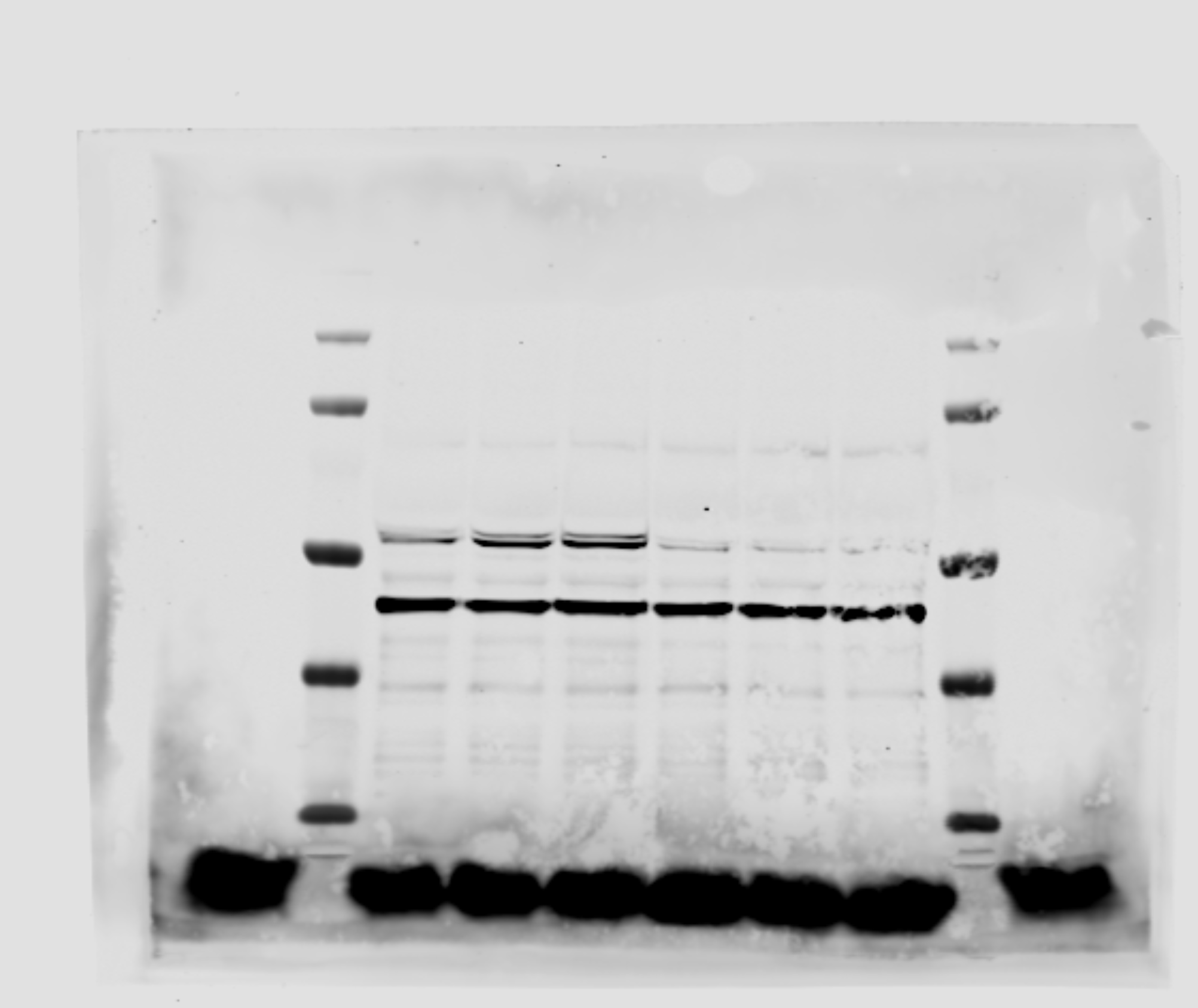

Supplement: Figure 1—source data 1. — Size of molecular weight (MW) markers are indicated. Bands for PTBP1 (57 kDa) and beta-actin (45 kDa) are indicated for sgRNA-ex3 in N2a and C8-D1A cells. [file elife-97180-fig1-data1.zip › Figure1SourceData1/Neuro2a-PTBP1.tif]

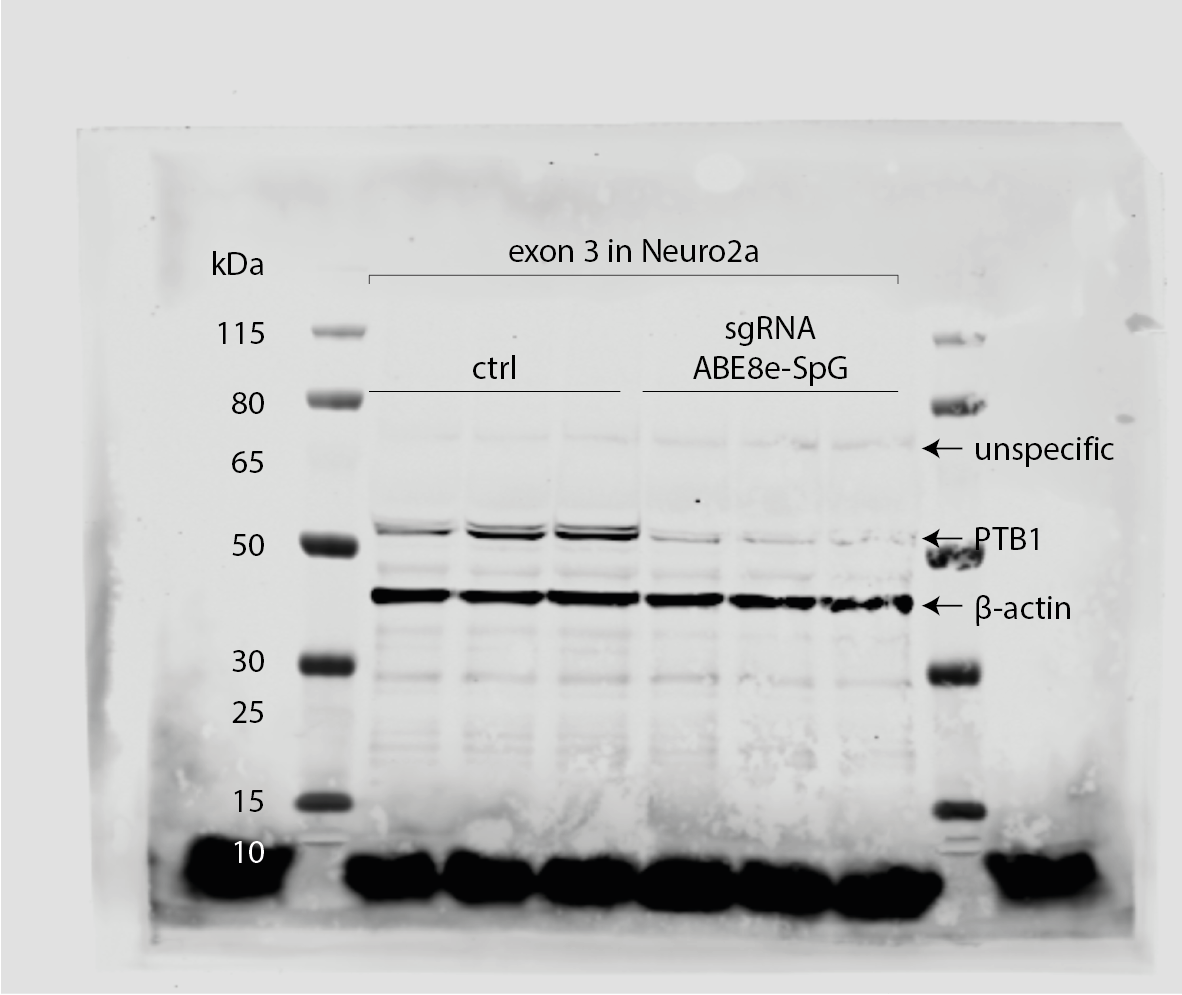

Supplement: Figure 1—source data 1. — Size of molecular weight (MW) markers are indicated. Bands for PTBP1 (57 kDa) and beta-actin (45 kDa) are indicated for sgRNA-ex3 in N2a and C8-D1A cells. [file elife-97180-fig1-data1.zip › Figure1SourceData1/Neuro2a-PTBP1_annotated.tif]

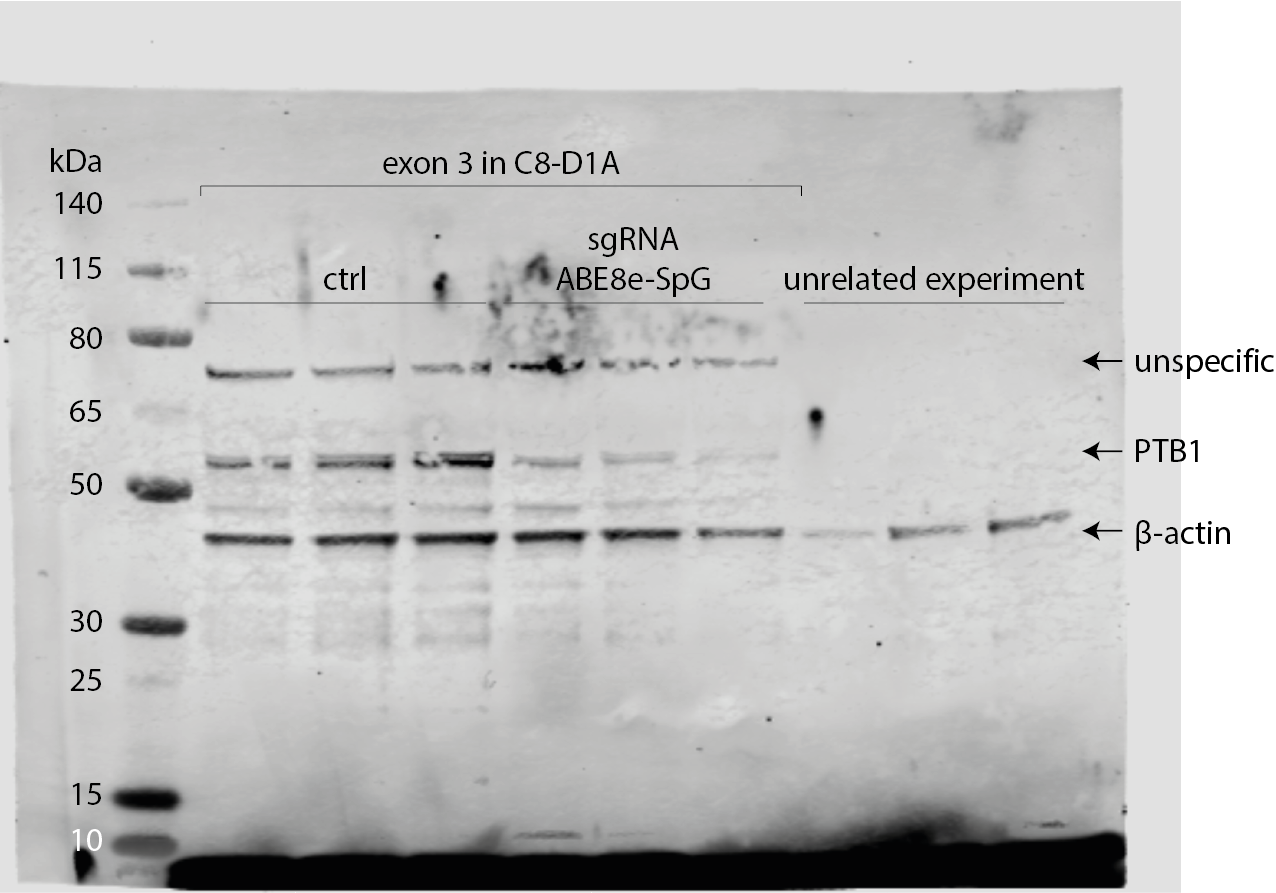

Supplement: Figure 1—source data 1. — Size of molecular weight (MW) markers are indicated. Bands for PTBP1 (57 kDa) and beta-actin (45 kDa) are indicated for sgRNA-ex3 in N2a and C8-D1A cells. [file elife-97180-fig1-data1.zip › Figure1SourceData1/C8D1A-PTBP1-repetition-revision_annotated.tif]

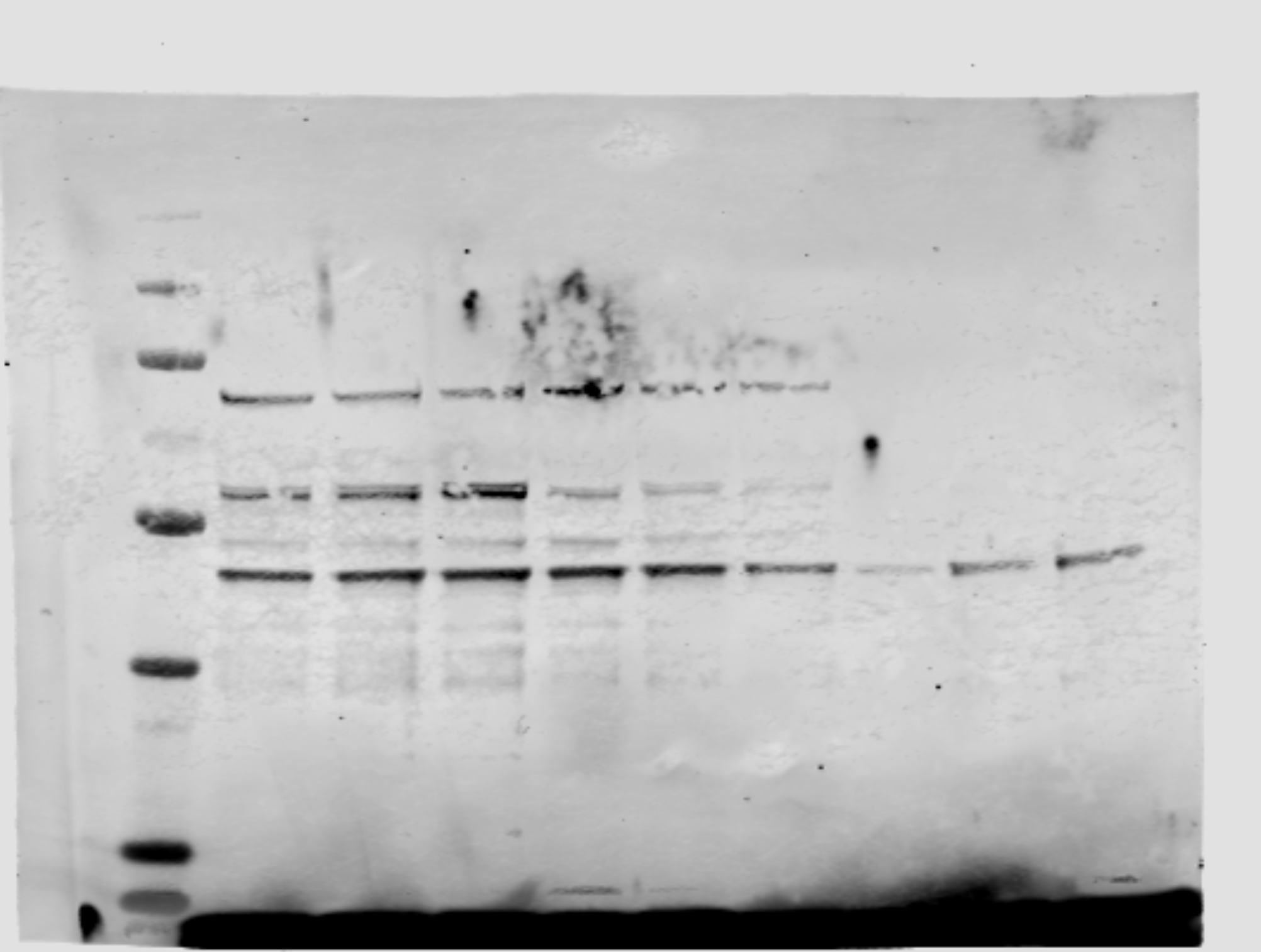

Supplement: Figure 1—source data 1. — Size of molecular weight (MW) markers are indicated. Bands for PTBP1 (57 kDa) and beta-actin (45 kDa) are indicated for sgRNA-ex3 in N2a and C8-D1A cells. [file elife-97180-fig1-data1.zip › Figure1SourceData1/C8D1A-PTBP1-repetition-revision.tif]

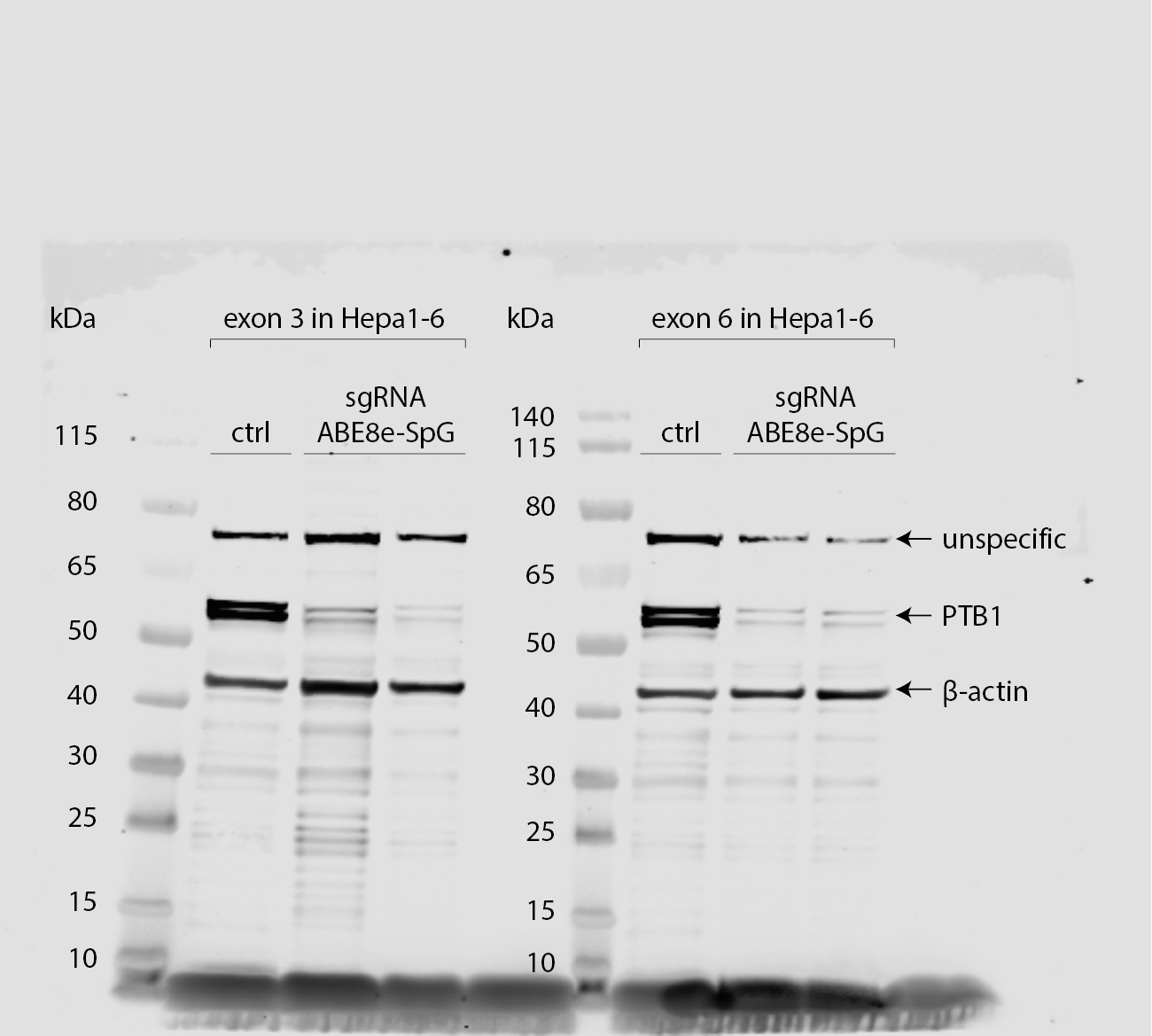

Supplement: Figure 1—figure supplement 2—source data 1. — Size of molecular weight (MW) markers are indicated. Bands for polypyrimidine tract binding protein 1 (PTBP1) (57 kDa) and beta-actin (45 kDa) are indicated for sgRNA-ex3 and sgRNA-ex7 in Hepa1-6 cells. [file elife-97180-fig1-figsupp2-data1.zip › Figure1FigureSupplement2SourceData1/Hepa-PTBP1_annotated.tif]

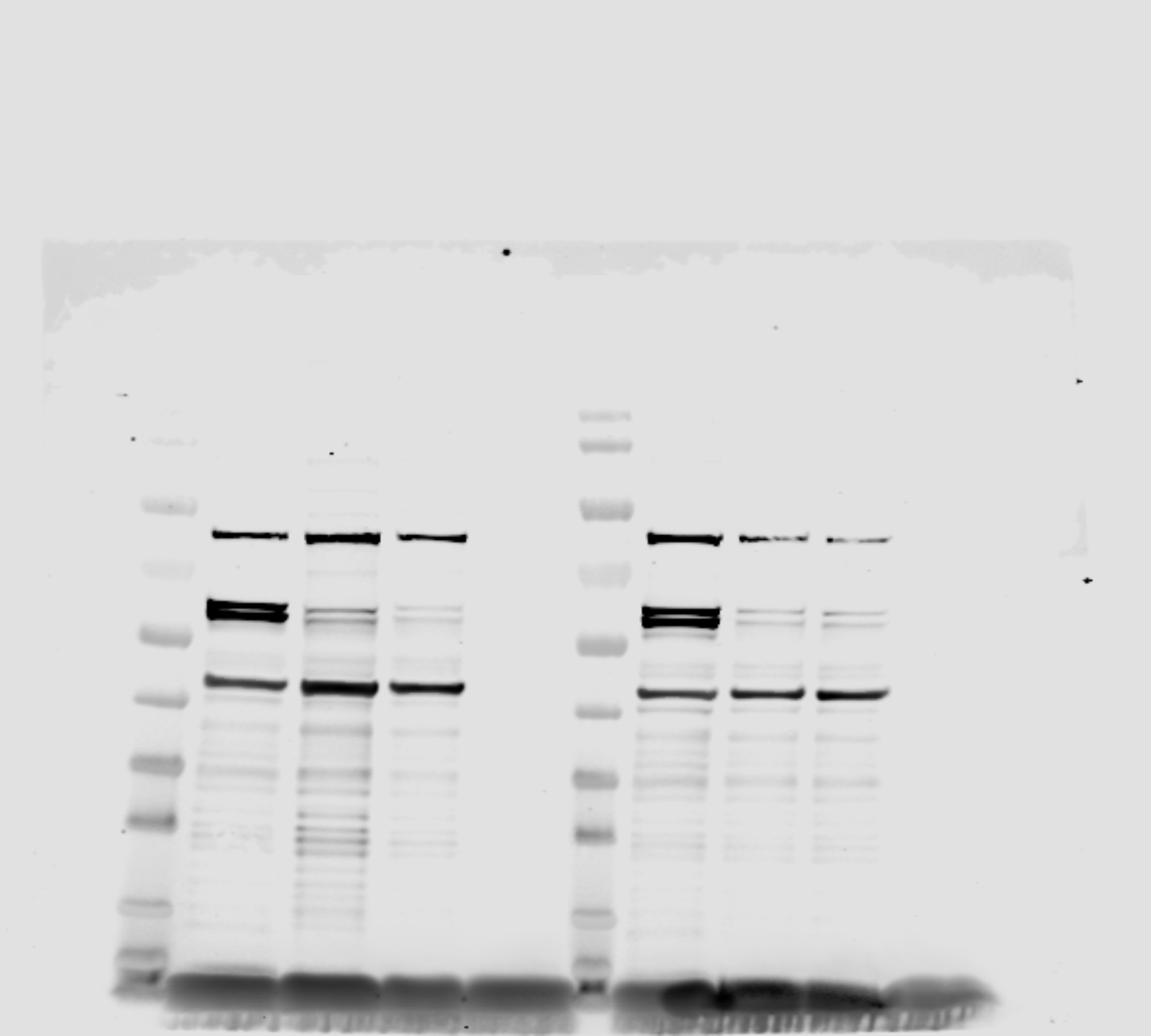

Supplement: Figure 1—figure supplement 2—source data 1. — Size of molecular weight (MW) markers are indicated. Bands for polypyrimidine tract binding protein 1 (PTBP1) (57 kDa) and beta-actin (45 kDa) are indicated for sgRNA-ex3 and sgRNA-ex7 in Hepa1-6 cells. [file elife-97180-fig1-figsupp2-data1.zip › Figure1FigureSupplement2SourceData1/HEPA-PTBP1.tif]

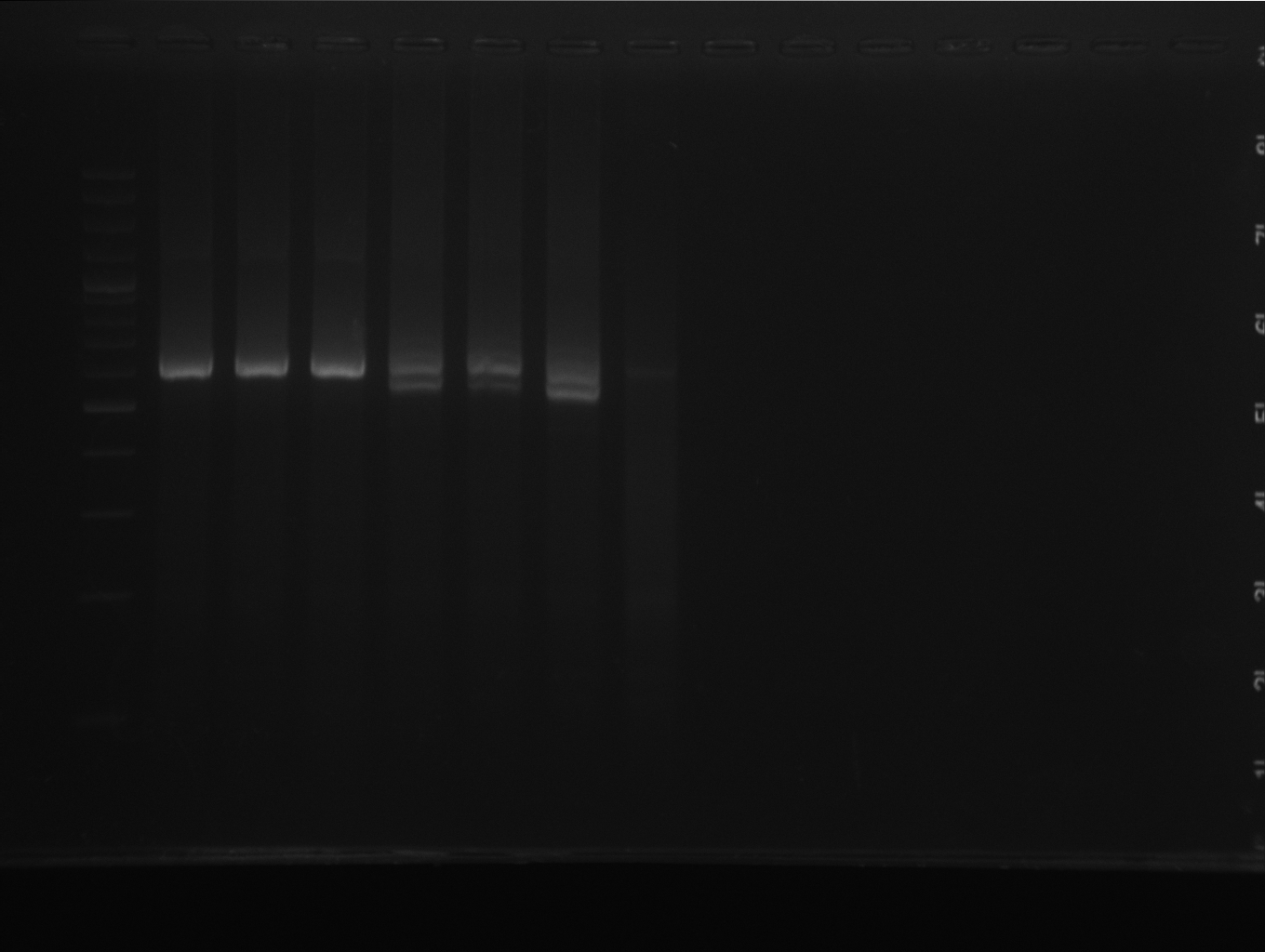

Supplement: Figure 1—figure supplement 3—source data 1. — Size of molecular weight (MW) markers are indicated. Bands for correctly spliced isoforms are indicated. [file elife-97180-fig1-figsupp3-data1.zip › Figure1FigureSupplement3SourceData1/cDNA-C8D1A.Tif]

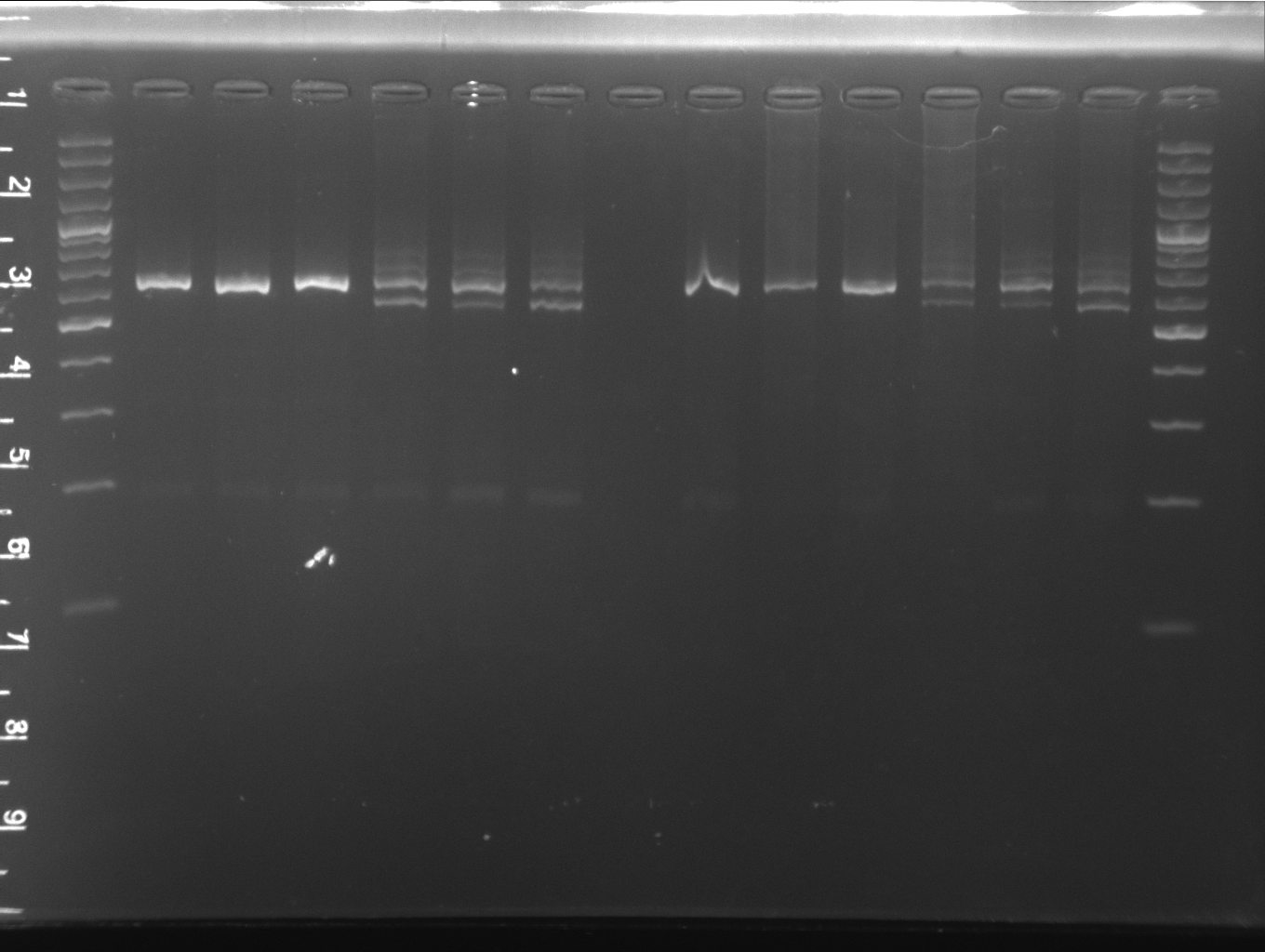

Supplement: Figure 1—figure supplement 3—source data 1. — Size of molecular weight (MW) markers are indicated. Bands for correctly spliced isoforms are indicated. [file elife-97180-fig1-figsupp3-data1.zip › Figure1FigureSupplement3SourceData1/cDNA-Neuro2A.Tif]

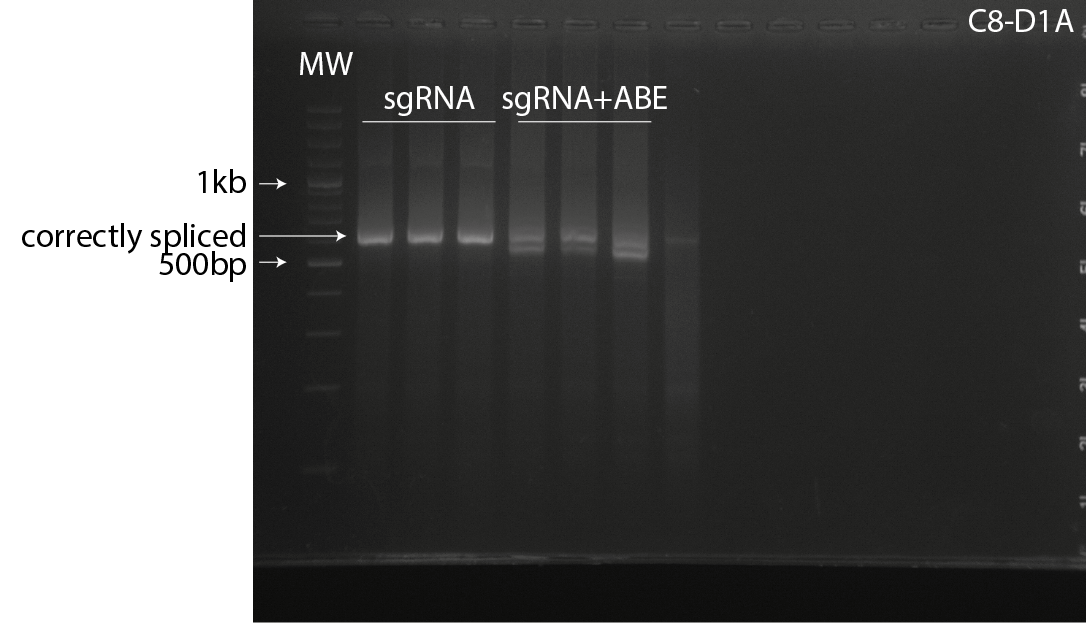

Supplement: Figure 1—figure supplement 3—source data 1. — Size of molecular weight (MW) markers are indicated. Bands for correctly spliced isoforms are indicated. [file elife-97180-fig1-figsupp3-data1.zip › Figure1FigureSupplement3SourceData1/cDNA-C8D1A_annotated.tif]

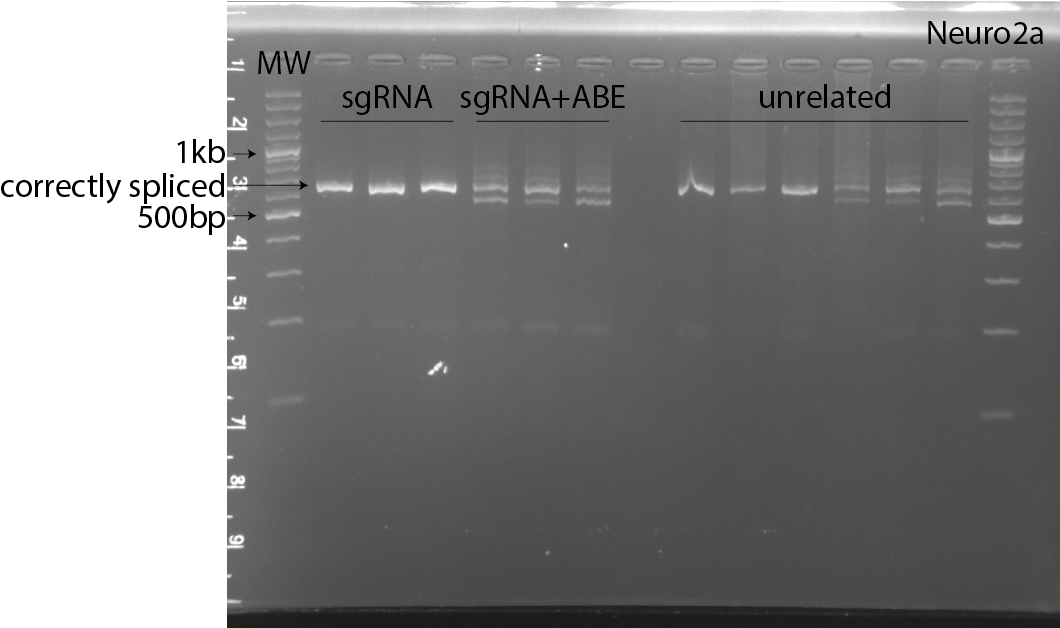

Supplement: Figure 1—figure supplement 3—source data 1. — Size of molecular weight (MW) markers are indicated. Bands for correctly spliced isoforms are indicated. [file elife-97180-fig1-figsupp3-data1.zip › Figure1FigureSupplement3SourceData1/cDNA-Neuro2A_annotated.tif]

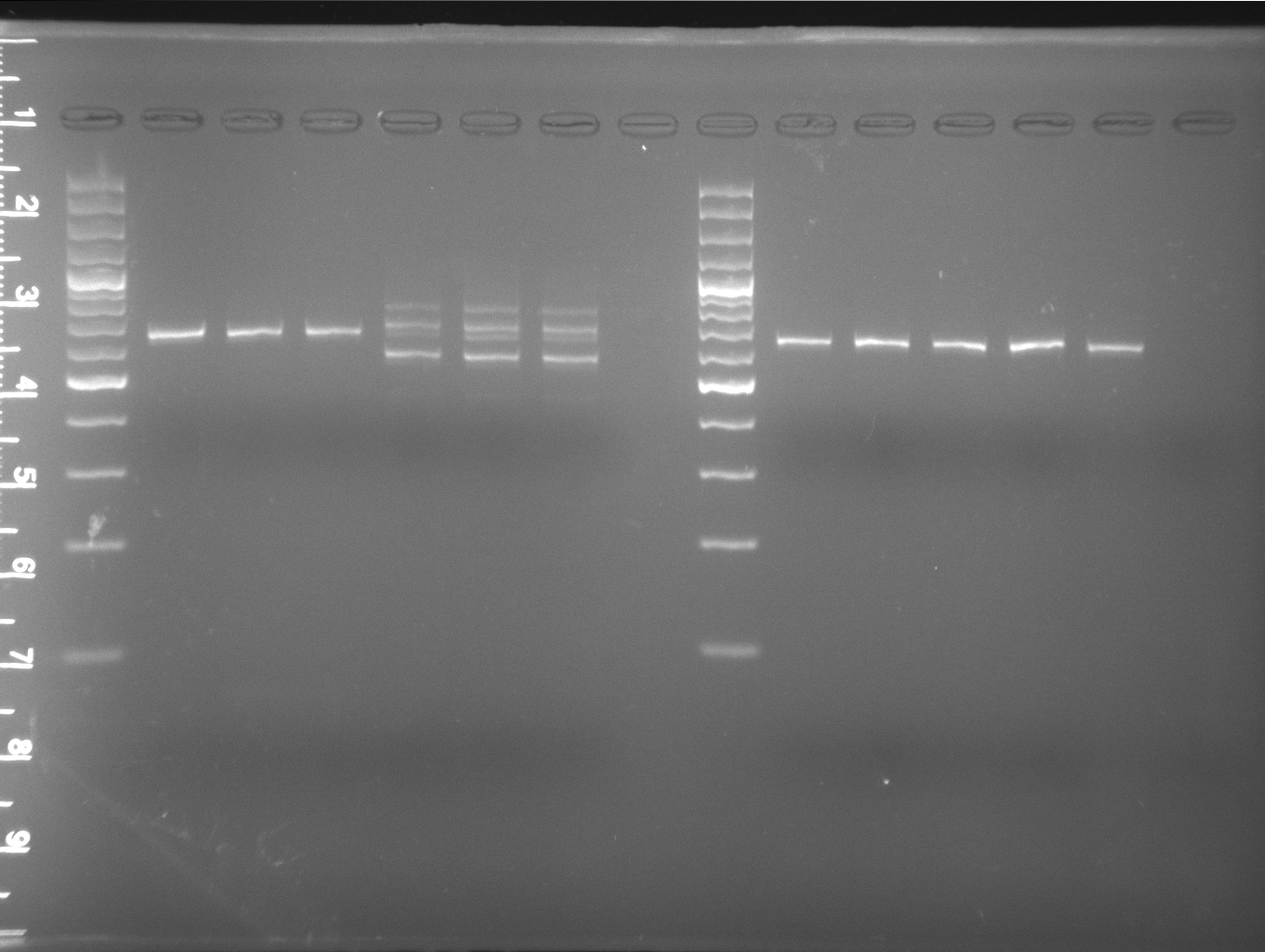

Supplement: Figure 1—figure supplement 3—source data 1. — Size of molecular weight (MW) markers are indicated. Bands for correctly spliced isoforms are indicated. [file elife-97180-fig1-figsupp3-data1.zip › Figure1FigureSupplement3SourceData1/cDNA-HEPA.Tif]

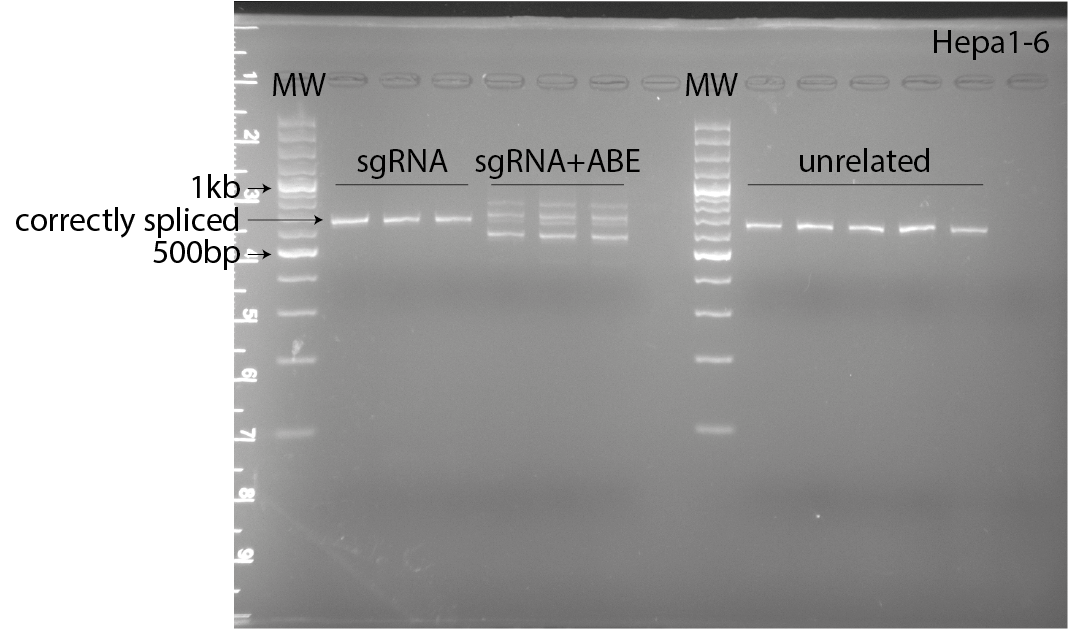

Supplement: Figure 1—figure supplement 3—source data 1. — Size of molecular weight (MW) markers are indicated. Bands for correctly spliced isoforms are indicated. [file elife-97180-fig1-figsupp3-data1.zip › Figure1FigureSupplement3SourceData1/cDNA-HEPA_annotated.tif]

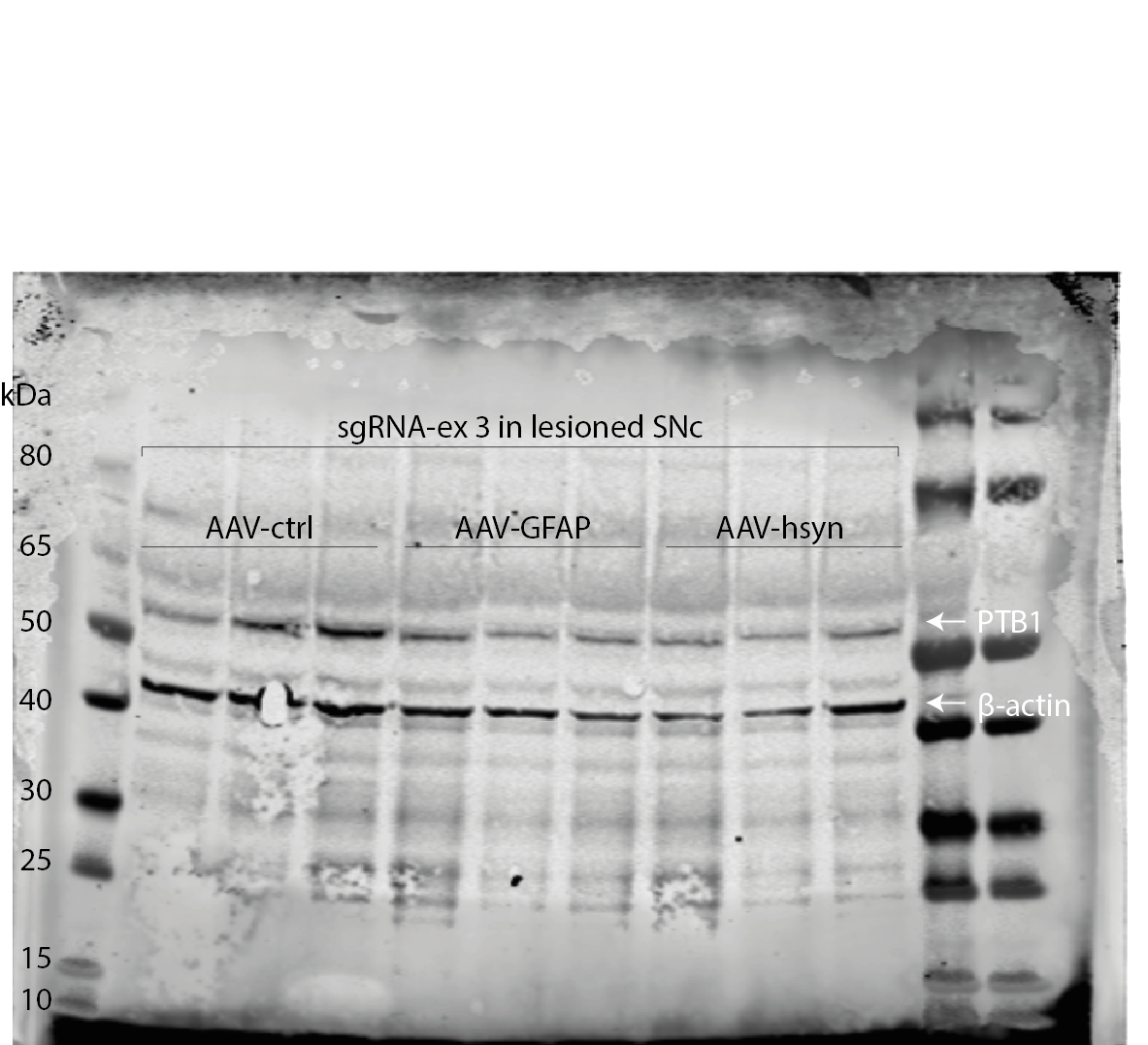

Supplement: Figure 2—figure supplement 5—source data 1. — Size of molecular weight (MW) markers are indicated. Bands for polypyrimidine tract binding protein 1 (PTBP1) (57 kDa) and beta-actin (45 kDa) are indicated in the substantia nigra pars compacta (SNc) of Parkinson’s disease (PD) mice. [file elife-97180-fig2-figsupp5-data1.zip › Figure2FigureSupplement5SourceData1/SNc-PTBP1_PDmice-revisions_annotated.tif]

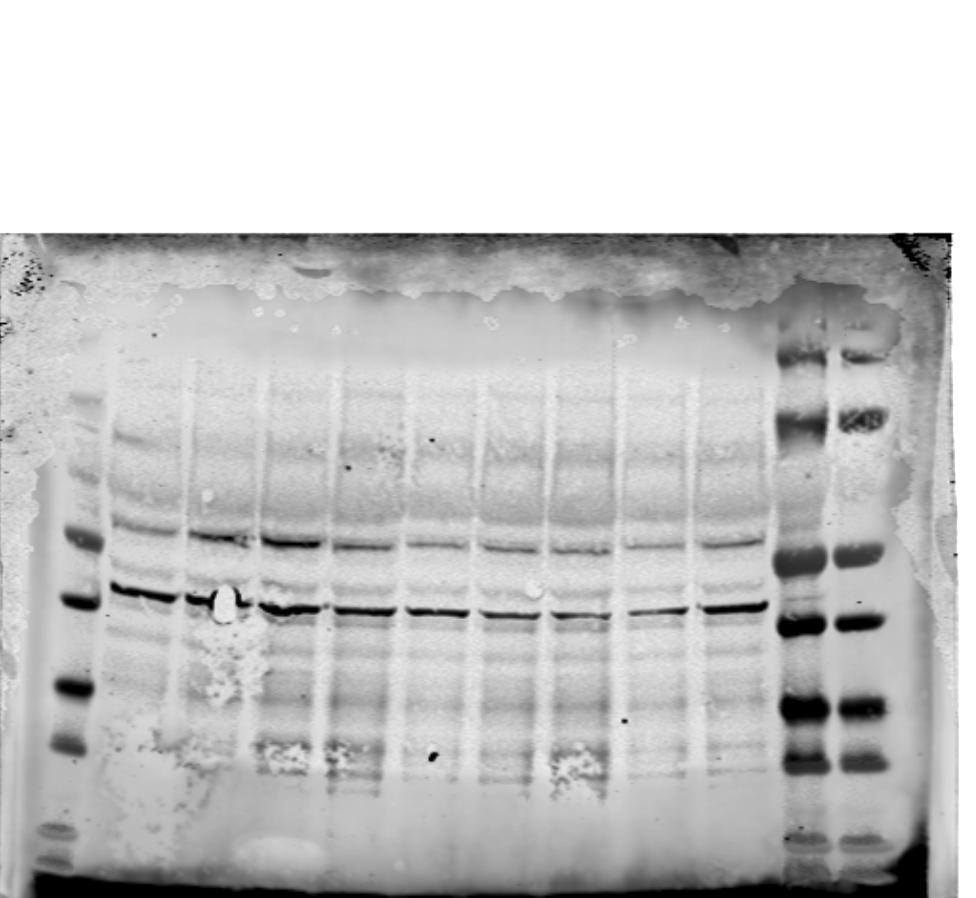

Supplement: Figure 2—figure supplement 5—source data 1. — Size of molecular weight (MW) markers are indicated. Bands for polypyrimidine tract binding protein 1 (PTBP1) (57 kDa) and beta-actin (45 kDa) are indicated in the substantia nigra pars compacta (SNc) of Parkinson’s disease (PD) mice. [file elife-97180-fig2-figsupp5-data1.zip › Figure2FigureSupplement5SourceData1/SNc-PTBP1_PDmice-revisions.tif]

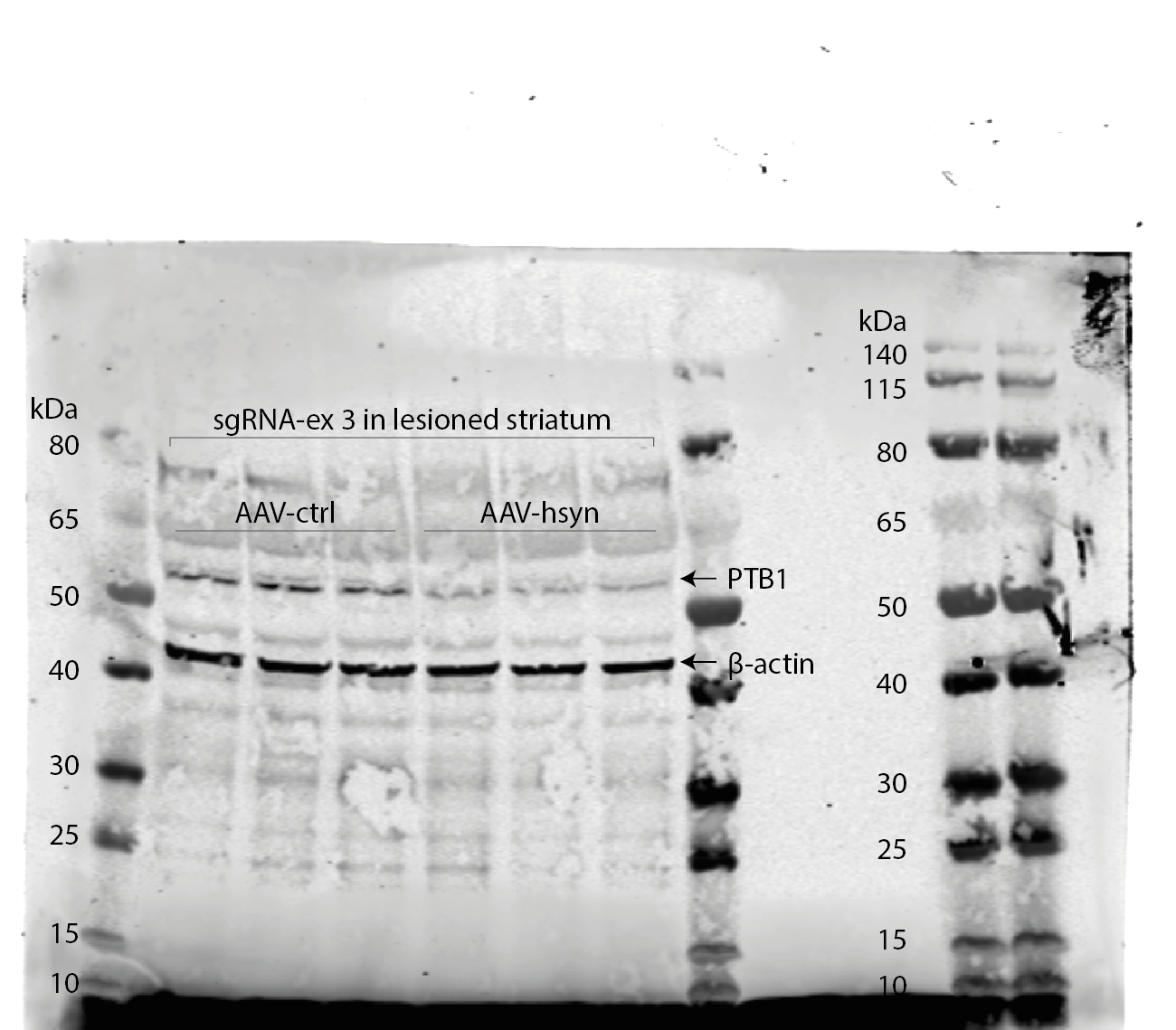

Supplement: Figure 3—figure supplement 2—source data 1. — Size of molecular weight (MW) markers are indicated. Bands for polypyrimidine tract binding protein 1 (PTBP1) (57 kDa) and beta-actin (45 kDa) are indicated in the striatum of Parkinson’s disease (PD) mice. [file elife-97180-fig3-figsupp2-data1.zip › Figure3FigureSupplement2SourceData1/striatum-PTBP1_PDmice-revisions_annotated.tif]

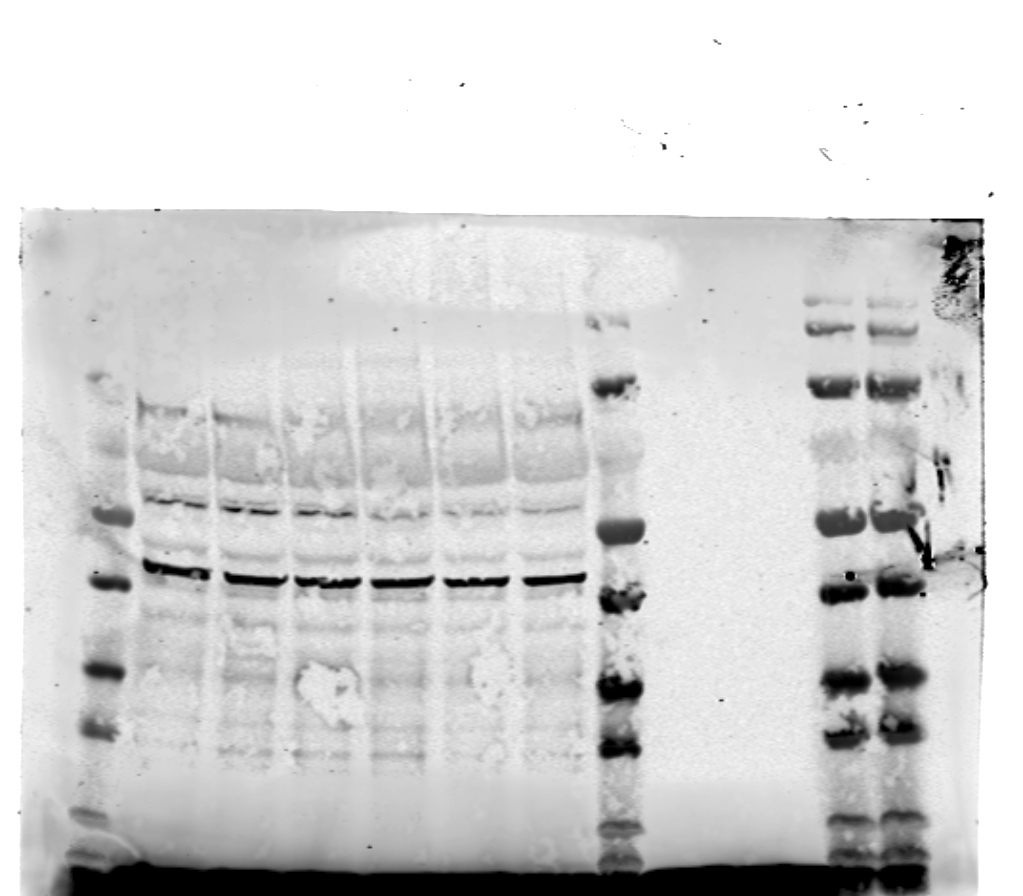

Supplement: Figure 3—figure supplement 2—source data 1. — Size of molecular weight (MW) markers are indicated. Bands for polypyrimidine tract binding protein 1 (PTBP1) (57 kDa) and beta-actin (45 kDa) are indicated in the striatum of Parkinson’s disease (PD) mice. [file elife-97180-fig3-figsupp2-data1.zip › Figure3FigureSupplement2SourceData1/striatum-PTBP1_PDmice-revisions.tif]
