# Supplementary material for: Base editing of Ptbp1 in neurons alleviates symptoms in a mouse model of Parkinson’s disease
Source: eLife. 2024 Dec 23;13:RP97180. doi: 10.7554/eLife.97180 (PMC11666242; doi:10.7554/eLife.97180)
Supplement: Supplementary file 1. [file elife-97180-supp1.docx]

Supplementary File 1

| oligo name | sgRNA_ID | sequence (5’ 🡪 3’) |
| --- | --- | --- |
| sgRNA01_PTBP1_fwd | sgRNA-01 | CACCGACTTACCCGTCCATGGCACA |
| sgRNA01_PTBP1_rev |  | AAACTGTGCCATGGACGGGTAAGTC |
| sgRNA02_PTBP1_fwd | sgRNA-02 | CACCGCTTACCTGCTGAGGCAGAGC |
| sgRNA02_PTBP1_rev |  | AAACGCTCTGCCTCAGCAGGTAAGC |
| sgRNA03_PTBP1_fwd | sgRNA-03 | CACCGTTCTCAGCGGGGATCCGACG |
| sgRNA03_PTBP1_rev |  | AAACCGTCGGATCCCCGCTGAGAAC |
| sgRNA04_PTBP1_fwd | sgRNA-04 | CACCGACTCACCAGCTTGGCATGCT |
| sgRNA04_PTBP1_rev |  | AAACAGCATGCCAAGCTGGTGAGTC |
| sgRNA05_PTBP1_fwd | sgRNA-05 | CACCGCCCACAGTCCCTGGATGGCC |
| sgRNA05_PTBP1_rev |  | AAACGGCCATCCAGGGACTGTGGGC |
| sgRNA06_PTBP1_fwd | sgRNA-06 | CACCGCTTACCAAAGGCTGCTGCCA |
| sgRNA06_PTBP1_rev |  | AAACTGGCAGCAGCCTTTGGTAAGC |
| sgRNA07_PTBP1_fwd | sgRNA-07 | CACCGAATACCTGCGGCCTGAGGGA |
| sgRNA07_PTBP1_rev |  | AAACTCCCTCAGGCCGCAGGTATTC |
| sgRNA08_PTBP1_fwd | sgRNA-08 | CACCGACATACCTCAGGGTTCAGAT |
| sgRNA08_PTBP1_rev |  | AAACATCTGAACCCTGAGGTATGTC |
