# Supplementary material for: Base editing of Ptbp1 in neurons alleviates symptoms in a mouse model of Parkinson’s disease
Source: eLife. 2024 Dec 23;13:RP97180. doi: 10.7554/eLife.97180 (PMC11666242; doi:10.7554/eLife.97180)
Supplement: Supplementary file 2. [file elife-97180-supp2.docx]

Supplementary File 2

| oligo name | sequence (5’ 🡪 3’) |
| --- | --- |
| pGfap-AAV-fwd | CGGCCTCTAGATCAGGGTACCAACATATCCTGGTGTGGAGTAGGG |
| pGfap-AAV-rev | CGGCCTCTAGATCAGGGTACCAACATATCCTGGTGTGGAGTAGGG |
| phsyn-AAV-fwd | CGGCCTCTAGATCAGGGTACCGAGGGCCCTGCGTATGAG |
| phsyn-AAV-rev | CTGTCCGTTTCATGGTGGCACCGGTCCAACTCTCGACTGCGCTC |
