# Supplementary material for: Base editing of Ptbp1 in neurons alleviates symptoms in a mouse model of Parkinson’s disease
Source: eLife. 2024 Dec 23;13:RP97180. doi: 10.7554/eLife.97180 (PMC11666242; doi:10.7554/eLife.97180)
Supplement: Supplementary file 3. [file elife-97180-supp3.docx]

Supplementary File 3

| oligo name | sequence (5’ 🡪 3’) |
| --- | --- |
| Gapdh-RTqPCR_fwd | CATCACTGCCACCCAGAAGACTG |
| Gapdh-RTqPCR_rev | ATGCCAGTGAGCTTCCCGTTCAG |
| Ptbp1-RTqPCR_fwd | CACCGCTTCAAGAAACCAGGCT |
| Ptbp1-RTqPCR_rev | GTTGCTGGAGAAGAGGCTCTTG |
| Ptbp2-RTqPCR_fwd | CCTGTAACACTTGATGTCCTTCAC |
| Ptbp2-RTqPCR_rev | CACCATACTGGAGCAAAGCCTG |
| Ptbp3-RTqPCR_fwd | CTCGCTGGTTTCCCGGAG |
| Ptbp3-RTqPCR_rev | TCCCCGCTTTAAACCGACTG |
| Kcnq2-RTqPCR_fwd | GTCTTCTCCTGCCTTGTGCT |
| Kcnq2-RTqPCR_rev | GCAGCCCAGATCCTCACAAA |
| pGfap-AAV-fwd | CGGCCTCTAGATCAGGGTACCAACATATCCTGGTGTGGAGTAGGG |
| pGfap-AAV-rev | CGGCCTCTAGATCAGGGTACCAACATATCCTGGTGTGGAGTAGGG |
| phsyn-AAV-fwd | CGGCCTCTAGATCAGGGTACCGAGGGCCCTGCGTATGAG |
| phsyn-AAV-rev | CTGTCCGTTTCATGGTGGCACCGGTCCAACTCTCGACTGCGCTC |
