# Supplementary material for: Base editing of Ptbp1 in neurons alleviates symptoms in a mouse model of Parkinson’s disease
Source: eLife. 2024 Dec 23;13:RP97180. doi: 10.7554/eLife.97180 (PMC11666242; doi:10.7554/eLife.97180)
Supplement: Supplementary file 4. [file elife-97180-supp4.docx]

Supplementary File 4

| antibody | clone | host species | dilution | application |
| --- | --- | --- | --- | --- |
| NEUN | EPR12763 | rabbit | 1:1’000 | histology, primary |
| GFAP | ab95231 | chicken | 1:1’500 | histology, primary |
| TH | na | mouse | 1:1’000 | histology&4i, primary |
| TH | ab76442 | chicken | 1:500 | histology, primary |
| *Sp*Cas9 | 7A9-3A3 | mouse | 1:50 | histology, primary |
| BrdU | BU1/75 (ICR1) | rat | 1:400 | histology, primary |
| DCX | sc-8066 | goat | 1:300 | 4i, primary |
| Nestin | EPR22023 | rabbit | 1:100 | 4i, primary |
| Sox2 | 14-9811-82 | rat | 1:300 | 4i, primary |
| DAT | ab184451 | rabbit | 1:300 | 4i, primary |
| CTIP2 | 25B6 | rat | 1:500 | 4i, primary |
| PV | EPR13091 | goat | 1:100 | 4i, primary |
| SST | G10 | mouse | 1:250 | 4i, primary |
| CALB2 | 6B3 | mouse | 1:250 | 4i, primary |
| anti-rabbit AF488 | JIR-711-545-152 | donkey | 1:1’000 | histology, secondary |
| anti-rabbit Cy3 | JIR-711-165-152 | donkey | 1:500 | histology, secondary |
| anti-chicken Cy5 | JIR-703-175-155 | donkey | 1:500 | histology, secondary |
| anti-mouse Cy3 | JIR-715-165-151 | donkey | 1:500 | histology, secondary |
| anti-goat AF488 | JIR-705-545-003 | donkey | 1:1’000 | histology, secondary |
| anti-goat Cy5 | JIR-705-175-147 | donkey | 1:5000 | histology, secondary |
| anti-rat Cy5 | JIR-712-175-153 | donkey | 1:500 | histology, secondary |
| PTBP1 | EPR9048B | rabbit | 1:10’000 | Western blot, primary |
| ACTB | ab8226 | mouse | 1:2’000 | Western blot, primary |
| IRDye® 680RD anti-rabbit IgG | 926-68073 | donkey | 1:20’000 | Western blot, secondary |
| IRDye® 800CW anti-mouse IgG | 926-32212 | donkey | 1:20’000 | Western blot, secondary |
