# Supplementary material for: Base editing of Ptbp1 in neurons alleviates symptoms in a mouse model of Parkinson’s disease
Source: eLife. 2024 Dec 23;13:RP97180. doi: 10.7554/eLife.97180 (PMC11666242; doi:10.7554/eLife.97180)
Supplement: Supplementary file 5. [file elife-97180-supp5.docx]

Supplementary File 5

| oligo name | sequence (5’ 🡪 3’) |
| --- | --- |
| PTBP1.ex1-HTS_fwd | CTTTCCCTACACGACGCTCTTCCGATCTNNNNNNTTCTGCTATTCCTGCGCCTC |
| PTBP1.ex1-HTS_rev | GGAGTTCAGACGTGTGCTCTTCCGATCTNNNNNNCATTGCAGCGGTTAGAGGGA |
| PTBP1.ex3-HTS_fwd | CTTTCCCTACACGACGCTCTTCCGATCTNNNNNNTGCAAATGGGAATGCAGGAA |
| PTBP1.ex3-HTS_rev | GGAGTTCAGACGTGTGCTCTTCCGATCTNNNNNNCAGGTCCTTTCTCCCAGCTC |
| PTBP1.ex7.1-HTS_fwd | CTTTCCCTACACGACGCTCTTCCGATCTNNNNNNATGCCAAGCTGGTGAGTAGG |
| PTBP1.ex7.1-HTS_rev | GGAGTTCAGACGTGTGCTCTTCCGATCTNNNNNNGCAGGTCAGGTCGAGTGTAG |
| PTBP1.ex7.2-HTS_fwd | CTTTCCCTACACGACGCTCTTCCGATCTNNNNNNCTACACTCGACCTGACCTGC |
| PTBP1.ex7.2-HTS_rev | GGAGTTCAGACGTGTGCTCTTCCGATCTNNNNNNATCACTGCACCTCACCTCAC |
| PTBP1.ex8-HTS_fwd | CTTTCCCTACACGACGCTCTTCCGATCTNNNNNNGTCAGCCTCTCCGTATGCAG |
| PTBP1.ex8-HTS_rev | GGAGTTCAGACGTGTGCTCTTCCGATCTNNNNNNCCTGTGGATGCTTCTGGAGG |
| PTBP1.ex9-HTS_fwd | CTTTCCCTACACGACGCTCTTCCGATCTNNNNNNGGTGCTGGGAATTCTGTCCT |
| PTBP1.ex9-HTS_rev | GGAGTTCAGACGTGTGCTCTTCCGATCTNNNNNNCAGCAGTGGCAGATAGAGGG |
| Mypn1_HTS_fwd | CTTTCCCTACACGACGCTCTTCCGATCTNNNNNNGGTCAAAAATGGCGCAAGGT |
| Mypn1_HTS_rev | GGAGTTCAGACGTGTGCTCTTCCGATCTNNNNNNTGCACACACTGACAAGGACT |
| Ank1_HTS_fwd | CTTTCCCTACACGACGCTCTTCCGATCTNNNNNNTGTTTTCTGCTTCTCAGGGGA |
| Ank1_HTS_rev | GGAGTTCAGACGTGTGCTCTTCCGATCTNNNNNNCAGTGGTCCAAGACCGTACA |
