# Supplementary material for: Base editing of Ptbp1 in neurons alleviates symptoms in a mouse model of Parkinson’s disease
Source: eLife. 2024 Dec 23;13:RP97180. doi: 10.7554/eLife.97180 (PMC11666242; doi:10.7554/eLife.97180)
Supplement: Supplementary file 6. [file elife-97180-supp6.docx]

Supplementary File 6

| amplicon name | amplicon sequence (5’🡪 3’) |
| --- | --- |
| PTBP1.ex1 | TTCTGCTATTCCTGCGCCTCCGCTCCGTTCCCCGCGGGTCTCTTCCGTGTGCCATGGACGGGTAAGTCCTGCCGCGCCCCTCGCACGCCGCTCCGCTCACCACTCCGTCCCAGCCATCGCTGCCGCGCGCGTGGACTTTTGGCCCCCGCCGATCCCTCTAACCGCTGCAATG |
| PTBP1.ex3 | TGCAAATGGGAATGCAGGAAAGGAATCAGCCTGGAACTAAGATTCCATGCTCTCTTCTCAGCGGGGATCCGACGAGCTCTTCTCCACGTGTGTCAGCAACGGCCCCTTCATCATGAGCAGCTCTGCCTCAGCAGGTAAGAGTGCCTGGGTGCCCTAGGGAGTCCTGCCTTGACAGGTACAGGGCGAGCTGGGAGAAAGGACCTG |
| PTBP1.ex7.1 | ATGCCAAGCTGGTGAGTAGGACTTGCTTGGGTGGGCAATCCTATGACTGGCCCACGCACTCACCTATGGCTCCCCACAGTCCCTGGATGGCCAGAACATCTACAACGCCTGCTGCACGCTGCGCATCGACTTCTCCAAGCTCACCAGTCTCAATGTCAAGTACAACAATGATAAGAGCAGAGACTACACTCGACCTGACCTGC |
| PTBP1.ex7.2 | CTACACTCGACCTGACCTGCCCTCTGGAGACAGCCAGCCTTCACTAGACCAGACCATGGCAGCAGCCTTTGGTAAGATGCTGTACTGAGACACACCAAATGAACAGGGGTGGGACAGGCCACCTAGCTGTCAGGGCACCCTGGCACTGCACAGCCCAGCCACTCAGCAGTCCTGCCCGTGCCTGGCCCAGCCGTGAGGTGAGGTGCAGTGAT |
| PTBP1.ex8 | GTCAGCCTCTCCGTATGCAGGAGCCGGGTTCCCTCCCACCTTTGCCATCCCTCAGGCCGCAGGTATTCACGCTCATCCTGACCCCAGCGCCTGCATGCCCACACAGCCCCATAACTGTCCATAGACCGGGATGCCACTGGCCCCAAGTGTTAGGCCCCAGGCCCCCTTCCTTCTGGGGAGAGGGGAAGGGGCCTCCAGAAGCATCCACAGG |
| PTBP1.ex9 | GGTGCTGGGAATTCTGTCCTTTTGGTCAGCAATCTGAACCCTGAGGTATGTGGGTATTGCTGTGCTCTGCTTATACATGGAGTAGTGGTGGGGTGCTGACTGACCACAAGTCAGGGTGGGGGAGCATACTGGATGGCAAGCAGGGTTTCTGGGTGTCCCAGGCGCCGTGTGGGTATAGGCGTGCTGCCCCTCTATCTGCCACTGCTG |
| Mypn-OT1 | GGTCAAAAATGGCGCAAGGTCAGAGGGCACCGTGGGTTCCAGCACACCTGCCCGCAGCGCTTCATCCATAGGCCCTGAGCTACCACCCAGCTCTGCTTCAGCAGGTACCCAGTACAGCTTGAGGTCAGCAGCTTGCTCCCATGCCTGTTCTTTGCTTTGCAAATGAGTCCTTGTCAGTGTGTGCA |
| Ank1-OT2 | TGTTTTCTGCTTCTCAGGGGAGAATCAGCTGTTCTGGCCATTTGATGGTACAGGGTTTGCCCATTCGAAGCCGACTCAGCCCTGCTCTGTCTCACAGGTAAGCACACGAGATCCCCCTTTTCTGGAGCCCTCCAGCGACCCCACTCACCACAGTGAACAGAAGTTGTACGGTCTTGGACCACTG |
